# Supplementary material for: Sub-national modelling of surveillance sensitivity to inform declaration of disease elimination: A retrospective validation against the elimination of wild poliovirus in Nigeria
Source: PLoS Comput Biol. 2026 Mar 16;22(3):e1013984. doi: 10.1371/journal.pcbi.1013984 (PMC13035237; doi:10.1371/journal.pcbi.1013984)
Supplement: Attachment — Submitted filename: review1.docx [file pcbi.1013984.s002.docx]

Review the manuscript: « Sub-national estimation of surveillance sensitivity to inform declaration of disease elimination: A retrospective validation against the elimination of wild poliovirus in Nigeria »

Submitted in POS Computational Biology

Manuscript number: PCOMPBIOL-D-25-00277

**Summary**

The authors present a statistical framework for estimating, month by month, the probability that Nigeria is now free of wild-type poliovirus serotype 1 (WPV1). Their model incorporates both acute flaccid paralysis (AFP) case data and environmental surveillance results. By explicitly accounting for the sensitivity of each surveillance system, the method yields an updated probability that WPV1 transmission has been interrupted.

The approach is potentially valuable: it could help other countries to assess when polio elimination is likely to have been achieved and therefore when immunisation campaigns might be relaxed.

Nonetheless, I noted several issues :

• Certain sections of the Methods lack clarity, making the model difficult to follow for readers unfamiliar with this approach.
• Some parameter values are introduced without justification.
• Additional sensitivity analyses are needed to provide a more reliable estimate of uncertainty.
• The manuscript shows occasional typographical and formatting errors that need careful proofreading.

Below is a detailed description of the improvements required.

**Major comments:**

**Material and methods:**

- Justify the choice of the regional design prevalence $D_{p}^{region}$=1/774 (774 being the number of LGAs). How does this align with the national design prevalence of 1 / 100,000?
- You assume a mean probability of notification of **0.9** (95 % CI 0.60–1.00) for an AFP case. Please cite at least one source, even from another country, to support this value.
- Provide a reference for the assumed diagnostic test sensitivity of **97 %**. The Supplementary Material calls this the “standard diagnostic test sensitivity,” but no citation is given.
- Cite a source for the assumed sensitivity of the laboratory assay used to detect poliovirus in environmental samples.
- Explain more clearly how the probability that an environmental sample is of adequate quality for poliovirus detection is calculated. In Supplementary Material, what do you mean by « Sampled values from this distribution are scaled with respect to the target rate of 50%, i.e. values less than 50% are scaled between 0 and 1 and values greater than 50% are assigned a value of 1. » ? Why are you targeting exactly 50% whereas **at least** 50% is consider to have a good surveillance system ?
- The model assumes that “all under-15s in the LGA are independent,” which is a strong simplification. Please discuss how violations of this independence assumption (such as clustering within households, schools, or neighbourhoods) could bias your results.

**Results:**

- Uncertainties of the sensitivity estimates of ENV surveillance are very small (Figure 5A, Figure 6A). How authors explain this? Would it not be more reasonable to incorporate more uncertainty?

**Discussion:**

- You argue that “a prior FFI probability around 0.5 could be considered optimistic if a positive was observed in the previous month” So I recommand to perform a senstivity analysis with respect to this parameter to show its impact on FFI.

**Minor comments:**

**Abstract:**

- Define the acronym « **WPV1 »** at first use in the abstract.

**Material and methods:**

- Section “Freedom From Infection” is not clear and should be rewritten.
  - Give the formula for $P[FFI]$. Is it $P\left[ FFI \right]=\frac{1-D_{p}}{1-S_{e}D_{p}}$ ?
  - What does it mean exactly that the the country overall has ongoing circulation?
- Please define “uncertainty interval”, do you mean “confidence interval”?
- p.11 you write “**certainty** intervals”, do you mean “uncertainty intervals” or “confidence intervals”?
- Avoid using “if” to introduce notation. Instead of writing “If $T^{-}$ denotes …”, write “Let $T^{-}$ denote …”.
- The negative predictive value (NPV) is the conditional probability that there is a true absence of infection $I^{-}$ given that the observed absence of infection $T^{-}$, i.e ${NPV=P(I^{-}|T}^{-})$. The authors made a mistake and wrote: ${NPV=P(T^{-}|I}^{-})$.
- “NPV” is not an “analogy”, it is defined by ${P(I^{-}|T}^{-})$
- State somewhere in the manuscript that Nigeria has **774 LGAs.**
- For better clarity, the authors should define sensitivity and specificity also with conditional probability when they are introduced for the first time in the subsection “Statistical framework”
- For greater precision, it should be specified that the link between NPV and Se, Sp is made by Bayes' formula
- When authors estimate the probability that an infected individual falls within the catchment area, are they assuming that the risk of infection is spatially homogeneous across the entire national population? If so, this assumption explicitly should be state explicitly.
- 1/500 > 1/5000, so authors should write : « with 95% quantile interval between 1/5,000 and 1/500 »
- A comma is missing for 1/5000, should be 1/5,000.

**Results :**

- Define « IQR » before using it for the first time.
- In the abstract you write « In contrast, we conclude a probability of **98% (97.9-98.7%)** by the time elimination of the serotype was officially declared in 2020. » whereas in the results you write : « the FFI probability that is attained by the official declaration of WPV elimination in August 2020 (41,42) is **98.2% (97.6-98.6%)**. » Why estimation of FFI at time of delcaration of elimination does not fully correspond in the Abstract and Results ?

**Figures/Tables:**

- Table 1:
  - The heading “95 % certainty interval” should read **“95 % uncertainty interval”** (or “95 % confidence interval”).
  - The confidence interval for the probability of notification is listed as 0.60–0.99, whereas the Supplementary Material gives 0.60–0.999. Please standardise. Note that 0.999 rounds to 1.00 (two decimal places), not 0.99.
  - Subscripts “i” are missing for $P^{Clinical}, P^{StoolTest}, P^{SampleTest}$
- Figure 2:
  - For greater clarity, please split the current Figure A into two panels and label them A and B (which means the present panel B will become C). In addition, state in the caption that ES = environmental samples.
