# Supplementary material for: Sub-national modelling of surveillance sensitivity to inform declaration of disease elimination: A retrospective validation against the elimination of wild poliovirus in Nigeria
Source: PLoS Comput Biol. 2026 Mar 16;22(3):e1013984. doi: 10.1371/journal.pcbi.1013984 (PMC13035237; doi:10.1371/journal.pcbi.1013984)
Supplement: Attachment — Submitted filename: Comments.docx [file pcbi.1013984.s003.docx]

Comments

This clearly written manuscript addresses a vexing epidemiological problem in a systematic and structured way. It is informed by previous modelling efforts and a real world context. The foundational epidemiological basis for the model is correct and the programmatic surveillance context and data are appropriately integrated into the model. The assumptions are valid and justified in the context of previous work. The findings are therefore robust and will contribute to the ongoing dialogue and recommendation development processes that are happening at a global level regarding cessation of bOPV2. This work should be published.

There are two possible areas for clarification, and a number of minor points.

Two areas for clarification

Re the numbers of people under environmental surveillance

- In the paragraph on page 14 (below Figure 3), the estimated population size within a 5km radius is 169,000. If the overall population density of Nigeria is 261/km2 (Wikipedia), and the area of a circle with radius 5km is 78km2, then the population within a 5km radius of the ES = 261x78=20,358 persons. If each ES site is designed to collect samples from c. 100,000 persons, this implies a population density of 1,282 persons/km2 within a 5km radius of each ES sampling site. This seems unrealistic.
- The figures of 169,000 persons within a catchment radius of 5km (on p 14, as above) appears to contradict the figures given in the discussion on page 20, paragraph 1 ‘For environmental surveillance….’ where by implication a LGA with a single ES site has only 12,500 people under surveillance.
- Is it possible to use the actual numbers/proportions of persons under surveillance in Nigeria, rather than estimate them. I think actual numbers based on topographical and GIS data are available and used by the polio ES programme through Novel-T software (ES.world). The authors have stated in the discussion that ‘this work is yet to be adequately linked to sample locations’ – is this still the case amongst Nigerian ES sites?

Re the application of the model in other contexts

- Would it be illustrative to take the model a step further by serially halving and serially doubling (or decreasing/increasing by some other reasonable factor) the surveillance performance data inputs and determining how this impacts on the probability of correctly determining FFI in the Nigerian context?
- How exactly may the model be applied in real-world contexts at national level particularly with regard to declaring a VDPV2 outbreak over. How would the required data differ, or how could the model be strengthened to make it more context-specific?
- What does the model tell us about the WP1 outbreak in Mozambique/Malawi in 2021-2? Is it over? Or could we expect WP1 to be circulating in the region today?

Other minor comments/thoughts

1. A semantic/nomenclature type question – the ‘design prevalence’ (in the section ‘Freedom from Infection’) implies that the polio surveillance system, comprising AFP and ES data has been ‘designed’ to meet a specific prevalence requirement that is required to interrupt transmission. Would it not be more correct to call it the ‘System prevalence’ or the ‘Minimum detectable prevalence’? If I understand it correctly – it is the lowest prevalence which the system is capable of detecting? The name ‘design prevalence’ seems tautologous, given that the sensitivity of the surveillance system could not be designed when it was envisaged and set up
2. LGA is used for the first time without defining the abbreviation in the section ‘Freedom from infection’ (it is define d later in the text)
3. Is there a basis for setting the ‘design prevalence’ at the level of 1/774 for the country, and for starting with the estimate of 1/100,000 ? is there evidence to suggest this? Given the discussion point that the conclusions are highly sensitive to the ‘design prevalence’, it may by helpful to explain the values the models started with – if it is possible to do so, without creating a circular argument (ie using the model outcome to justify the starting point!)
4. The estimates in table 1 are not referenced.
5. In the para ‘FFI probability over time’, the second paragraph begins with the word ‘Specifically’. I think this word is used for its linguistic meaning here, rather than its statistical meaning. So it may read better (in terms of meaning) to start with ‘Therefore…’ or some other way of transitioning to a more detailed explanation.
6. Map D in Figure 4 could be enlarged for easier readability
7. In the Discussion, in the section on ‘our estimation of AFP sensitivity’ – patient level (care-seeking) and health system level factors will impact the probability of a symptomatic case being reported. Conflict and social instability may impact both factors. In these settings, could all-cause mortality rates in U15s provide some context for interpreting or moderating the probability of AFP detection?
8. In the Discussion - the para starting ‘Having ES within sewage treatment works…’ can be contextualised by the fact that LMICs, and areas with highest risk for polio emergence have minimal wastewater infrastructure.
