# Supplementary material for: Sub-national modelling of surveillance sensitivity to inform declaration of disease elimination: A retrospective validation against the elimination of wild poliovirus in Nigeria
Source: PLoS Comput Biol. 2026 Mar 16;22(3):e1013984. doi: 10.1371/journal.pcbi.1013984 (PMC13035237; doi:10.1371/journal.pcbi.1013984)
Supplement: Attachment — Submitted filename: Response to reviewers.docx [file pcbi.1013984.s006.docx]

Faculty of Epidemiology and Population Health

London School of Hygiene and Tropical Medicine

Keppel Street, London, WC1E 7HT, U.K.

E-mail: emily.nightingale@lshtm.ac.uk

1^st^ September 2025

*Plos Computational Biology*

Dear Editor,

**RE: “Sub-national estimation of surveillance sensitivity to inform declaration of disease elimination”**

Thank you very much for the opportunity to revise and resubmit this manuscript. Please find below our responses to the reviewers’ comments.

Kind Regards,

Emily Nightingale

**Reviewer 1**
This is a well-written manuscript which seeks to quantify the sensitivity of Nigeria’s polio surveillance system and derive a statistical framework which provides a probability of freedom from infection (FFI). This is then retrospectively validated against two periods of empirical absence of WPV1. The principal findings from the framework are concordant with the true virus transmission status and aim to provide a quantitative, spatially specific decision aid in polio eradication scenarios.

I think this manuscript is suitable for publication in PLoS Comp. Biol. after a few minor revisions.

**Abstract**
1) The opening sentence is a bit clumsy and could probably do with being split into two, something like:
‘Which duration of absence of polio infection qualifies as regional elimination is a key question in the global drive towards eradication of the disease. The safe withdrawal of the polio vaccine is naturally contingent upon this.’

The sentence has been amended as suggested:

“A fundamental question in the global drive towards polio eradication is how long of a period without infection qualifies as regional elimination. The safe withdrawal of the oral polio vaccine is naturally contingent on the answer.”

2) Perhaps expand on ‘sensitivity over time and space’, introduce the time and spatial scales (as you have in the methods) more explicitly here so the reader can get a better feel for the size of the study.

Both temporal and spatial scales are now stated in the abstract:

[Abstract; lines 23-26]: “We present a statistical framework to model time-varying sensitivity of two key components of polio surveillance - environmental sampling and clinical cases of acute flaccid paralysis - for detecting infection on a monthly basis at the local government authority level.”

**Introduction**
1) The independence between cases of AFP and detection of the virus via ES has been assumed, is there a suitable reference for this? A couple of lines stating/explaining this, as you did with vaccination would be helpful.

A section has been added to the methods, results and discussion regarding assumed independence:

[Methods; lines 316-321]: This calculation makes a simplifying assumption of independence between the AFP and ENV systems. Due to the low symptomatic rate of polio infection, the overlap between infections detected via the environment and those having developed into AFP will be small, however correlation may be induced by external factors influencing both systems. This will be investigated via a simple correlation test of the estimated sensitivities.

[Results; lines 427-429]: A weak positive correlation is observed between AFP and ENV sensitivity per LGA and month during this period (Pearson’s correlation and 95% confidence interval: 0.28 [0.27-0.30]).

[Discussion; lines 557-563]: Inaccessibility is one of several external factors that could influence both AFP and ENV, potentially inducing correlation that violates the simplifying assumption of independence and would lead to over-estimation of sensitivity. Some evidence of positive correlation was observed between our estimates for the two systems on an LGA level. Accommodating this dependence could therefore slightly lengthen our estimated timelines for elimination, but not to an extent that would contradict our conclusions. The viability of this assumption is something that should be reconsidered for any new setting in which the approach is applied.

**Methods**
1) It would be good to include a summary of the sensitivity analyses you’ve conducted for some of the key assumptions (such as the design prevalence, prior FFI and ES catchment) in the main text rather than just stating you’ve done them. Please also refer to the specific item in the supplement (which for Plos comp biol is something like ‘Fig B in S1 Text’).

A section has been added in the main text to explain the sensitivity analyses:

[Results; lines 441-456]:

### Sensitivity analyses

We considered two alternative assumptions about catchment radius around sampling sites - 2km and 10km. Of course increasing the catchment size results in a larger estimated proportion of the population represented in ES and hence an increase in sensitivity. However, this difference was not large enough to have a substantial impact on the resulting elimination trajectory (*Supplementary Materials C; Figure S5*).

Specifying a less stringent design prevalence of 1 per 10,000 resulted in a high probability of freedom from infection as early as mid-2015 (*Supplementary Materials C; Figure S6*), which does not align with the known persistence of transmission during this time.

The assumed prior for the probability of freedom from infection immediately after the last observed positive does have a substantial influence on the inferred trajectory (*Supplementary Materials C; Figure S7*). In this example, assuming either an ambivalent or more conservative (i.e. lower) distribution resulted in estimates more consistent with the timeline that was observed.

2) Treating AFP and ES as independent will over-estimate combined sensitivity should these two variables be influenced by the same external factors, might be worth mentioning or a simple correlation test.

Please see response to similar comment (1) regarding the introduction.

3) Citation for lab test sensitivity?

This has been added:

[*Gerloff N, Sun H, Mandelbaum M, Maher C, Nix WA, Zaidi S, et al. Diagnostic Assay Development for Poliovirus Eradication. J Clin Microbiol. 2018 Feb;56(2):e01624-17*.](https://www.zotero.org/google-docs/?1GCLda)

4) Minor typo in ‘LGAs followers the approach’

Corrected.

**Results**
Figures look great and their message is clear. My only comment here is that it’s quite an ‘explosion of colour’ in some of the panels, perhaps describing the lines/colours in the legend could be helpful, particularly for colourblind readers.

The colour palettes used in all figures are interpretable with any type of colour blindness. Further description of the colours has been added to the legends:

[Figure 3; legend]: In all panels, performance is classified into three levels according to how consistently the WHO target threshold is met; completely (blue), mostly (green) and partially (red). LGAs are mapped by this classification in the two right-hand figures.

[Figure 4; legend]: As in Figure 3, performance in panel C is classified according to how consistently the WHO target threshold is met, here into four levels; completely (blue), mostly (green), partially (yellow) and not at all (red). LGAs are mapped by this classification in panel D.

[Figure 5; legend]: Estimated sensitivity of AFP (orange) and ENV (blue) surveillance per month, for detecting infection at the specified design prevalence of 1 per 100,000 in any LGA.

To reduce visual noise and avoid confusion with other panels, the colours in figure 4A have also been amended to a monochrome scale:

**Discussion**
1) Could contrast your findings more explicitly with Dietz and Eichner’s 3-year rule.

A comment on this comparison has been added to the discussion as suggested:

[Discussion; lines 466-72]: We further observe that, likely due to the expansion of ES after 2018, considering evidence from ES alone yields similar levels of confidence as pre-2018 data from the combined surveillance system. Our estimated trajectory for reaching the threshold of 95% is approximately two months shorter than Eichner and Dietz’s estimated timeline [(](https://www.zotero.org/google-docs/?TPBYkC)which did not incorporate evidence from environmental surveillance) - a difference that may be increased with greater ES coverage.

2) I think the suggestion of ES superseding AFP surveillance might need further caveating, they’re fundamentally different things and the ability of ES to supersede may not be relevant in all foci.

The sentence has been amended to avoid over-stating the contribution of ES:

[Discussion; lines 468-9]: This demonstrates the strength of ES relative to AFP surveillance in near-elimination settings.

3) The overall structure of the discussion gets a little jumpy and some of the paragraphs are long. Consider breaking it up into a more organised flow i.e. Implications – Limitations – Future work

Sub-headings of Key findings, Limitations, Implications for future research, and conclusions have been added to the discussion.

**Conclusion**
1) GCC not GGC in the opening line

Corrected.

2) A concise statement about broader generalisability outside of Nigeria (Pakistan, Afghanistan etc) could provide a nice way to strengthen the conclusion.

A sentence has been added as suggested:

[Conclusion; lines 592-7]: Using the approach demonstrated here, temporal changes and localised disruption to surveillance may directly and quantitatively inform our interpretation of the absence of detection, and the accumulating evidence that transmission has been interrupted. This inference may be extended to data from Pakistan and Afghanistan to serve WPV eradication goals, as well as regions of sub-Saharan Africa and Southeast Asia to determine the resolution of cVDPV2 outbreaks.

Reviewer 2

The authors present a statistical framework for estimating, month by month, the probability that Nigeria is now free of wild-type poliovirus serotype 1 (WPV1). Their model incorporates both acute flaccid paralysis (AFP) case data and environmental surveillance results. By explicitly accounting for the sensitivity of each surveillance system, the method yields an updated probability that WPV1 transmission has been interrupted.

The approach is potentially valuable: it could help other countries to assess when polio elimination is likely to have been achieved and therefore when immunisation campaigns might be relaxed.

Nonetheless, I noted several issues :

- Certain sections of the Methods lack clarity, making the model difficult to follow for readers unfamiliar with this approach.
- Some parameter values are introduced without justification.
- Additional sensitivity analyses are needed to provide a more reliable estimate of uncertainty.
- The manuscript shows occasional typographical and formatting errors that need careful proofreading.

Below is a detailed description of the improvements required.

**Major comments:**

**Material and methods:**

Justify the choice of the regional design prevalence<https://www.codecogs.com/eqnedit.php?latex=D_p%5E%7Bregion%7D#0> =1/774 (774 being the number of LGAs). How does this align with the national design prevalence of 1 / 100,000?

Further justification for these values had been given:

[Methods; lines 205-213]: We specify the design prevalence at two levels: LGA and country. There is not concrete evidence for a magnitude of infection prevalence that would sustain transmission, therefore we chose a value of<https://www.codecogs.com/eqnedit.php?latex=D_p%20%3D%201%2F100%2C000#0> as well as two less-stringent thresholds to explore in a sensitivity analysis. This was a pragmatic choice on the basis that the threshold should be at least less than 1/2,000 (due to the asymptomatic rate of WPV1) and conservative (due to the significant negative consequences of premature declaration). We then set a country-level, or regional, design prevalence,<https://www.codecogs.com/eqnedit.php?latex=D_p%5E%7Bregion%7D%20%3D%201%2F774#0> such that if prevalence persisted above<https://www.codecogs.com/eqnedit.php?latex=D_p#0> in any one of the 774 LGAs, we would not declare the country as a whole to be free from infection.

You assume a mean probability of notification of 0.9 (95 % CI 0.60–1.00) for an AFP case. Please cite at least one source, even from another country, to support this value.

The justification of this distribution in the supplementary materials has been expanded, with added citations:

[Supplementary Materials A; Case Notification]: AFP in children is a visible, debilitating condition that causes significant concern and motivates prompt care-seeking, as illustrated by a study from Ethiopia finding that 69% of parents sought care from a public health facility for their child with AFP [(3)](https://www.zotero.org/google-docs/?PUdKKd). In Nigeria during this period of time, there was intensive messaging around recognising and reporting AFP due to the WPV elimination efforts and target notification rates for surveillance [(4)](https://www.zotero.org/google-docs/?nFkzmu). We therefore work from the assumption that, where absolute AFP notification rates are high (i.e. meeting the WHO threshold), the probability of notification to a public health facility is high and at least more likely than not. The value is, however, uncertain due to unobserved barriers that exist between seeking and actually *accessing* the appropriate care. We therefore set a beta distribution for this parameter which predominantly extends between 0.6 and 0.99, with highest density at 0.9.

Provide a reference for the assumed diagnostic test sensitivity of 97 %. The Supplementary Material calls this the “standard diagnostic test sensitivity,” but no citation is given.

A citation has been added:

[*Gerloff N, Sun H, Mandelbaum M, Maher C, Nix WA, Zaidi S, et al. Diagnostic Assay Development for Poliovirus Eradication. J Clin Microbiol. 2018 Feb;56(2):e01624-17*.](https://www.zotero.org/google-docs/?1GCLda)

Cite a source for the assumed sensitivity of the laboratory assay used to detect poliovirus in environmental samples.

We refer to the same citation as above:

[*Gerloff N, Sun H, Mandelbaum M, Maher C, Nix WA, Zaidi S, et al. Diagnostic Assay Development for Poliovirus Eradication. J Clin Microbiol. 2018 Feb;56(2):e01624-17*.](https://www.zotero.org/google-docs/?1GCLda)

The assay is the same as that used for stool samples and the medium being tested is still stool. Poliovirus is stable in water for some time, but we added some further uncertainty to account for any minor impact of degradation.

Explain more clearly how the probability that an environmental sample is of adequate quality for poliovirus detection is calculated. In Supplementary Material, what do you mean by « Sampled values from this distribution are scaled with respect to the target rate of 50%, i.e. values less than 50% are scaled between 0 and 1 and values greater than 50% are assigned a value of 1. » ? Why are you targeting exactly 50% whereas at least 50% is considered to have a good surveillance system ?

This approach does not target exactly 50%. The explanation in the supplementary materials has been expanded to clarify this:

[Supplementary materials A; Sampling quality]: Sampled values from this distribution are scaled with respect to the threshold rate of 50%, i.e. values less than 50% are scaled between 0 and 1 while values greater than 50% are assigned a value of 1. In doing this, we are assuming that sensitivity is *maximised* with respect to sampling quality when this average rate at least meets the WHO threshold. The sensitivity in LGAs with lower rates of NPEV detection is then assumed to scale proportionally with how far below the threshold they fall.

The model assumes that “all under-15s in the LGA are independent,” which is a strong simplification. Please discuss how violations of this independence assumption (such as clustering within households, schools, or neighbourhoods) could bias your results.

This has been added as suggested:

[Discussion; lines 565-8]: Our conclusions are sensitive to some other key assumptions. First, the model accounts for dependence between under-15s within the same LGA, but not for clustering at a lower level (e.g. within schools). As with correlation between the two surveillance systems, this could result in over-estimation of sensitivity.

**Results:**

Uncertainties of the sensitivity estimates of ENV surveillance are very small (Figure 5A, Figure 6A). How authors explain this? Would it not be more reasonable to incorporate more uncertainty?

This difference in scale is now noted in the text:

[Results; lines 429-434]: A difference in scale arises from the substantially greater uncertainty in the probability that an AFP case is notified, compared to any of the stepwise probabilities relating to ES. The greatest source of uncertainty in ES sensitivity likely arises from catchment; however, through sensitivity analysis we find that relatively large uncertainties in the catchment area translate to small differences in sensitivity (*Supplementary Materials C; Figure S5*).

**Discussion:**

You argue that “a prior FFI probability around 0.5 could be considered optimistic if a positive was observed in the previous month” So I recommend to perform a sensitivity analysis with respect to this parameter to show its impact on FFI.

A sensitivity analysis of the prior FFI probability is already presented in the supplementary materials (Section C; Prior probability of FFI). This has now been referenced in the main text as part of a new section explaining the findings of the sensitivity analyses:

[Results; lines 447-451]: “The assumed prior for the probability of freedom from infection immediately after the last observed positive does have a substantial influence on the inferred trajectory (*Supplementary Materials C; Figure S7*). In this example, assuming either an ambivalent or more conservative (i.e. lower) distribution resulted in estimates more consistent with the timeline that was observed.”

**Minor comments:**

**Abstract:**

Define the acronym « WPV1 » at first use in the abstract.

Done:

[Introduction; lines 99-101]: Here, we apply and validate the estimation of regional freedom from wild poliovirus infection using the retrospective example of wild poliovirus serotype 1 (WPV1) elimination in Nigeria.

**Material and methods:**

Section “Freedom From Infection” is not clear and should be rewritten.

We would need further information about what specifically the reviewer finds unclear in this section in order to rewrite it. We hope that the revisions in response to comments listed here have improved the clarity overall and addressed this point.

Give the formula for P[FFI]. Is it<https://www.codecogs.com/eqnedit.php?latex=P%5BFFI%5D%20%3D%20%5Cfrac%7B1-D_p%7D%7B1-S_eD_p%7D#0>?

Apologies for the confusion regarding notation. The probability is denoted P_t^{Free} and defined in the equation on line 335 (Methods, Probability of freedom from infection):

<https://www.codecogs.com/eqnedit.php?latex=P%5E%7BFree%7D_%7Bt%2B1%7D%20%3D%20(1%20-%20P%5E%7BInfect%7D_%7Bt%7D)%2F(1-%20P%5E%7BInfect%7D_%7Bt%7D*Se_%7Bt%7D)%20#0>

The notation is now more clearly defined in the previous paragraph:

[Methods; lines 327-9]: The probability of FFI at time<https://www.codecogs.com/eqnedit.php?latex=t#0> (<https://www.codecogs.com/eqnedit.php?latex=P%5E%7BFree%7D_%7Bt%7D#0>) is defined as the probability that infection is below the design prevalence, given that no positive AFP cases or environmental samples have been observed in *any* LGA.

What does it mean exactly that the country overall has ongoing circulation?

With the revision above to the explanation of design prevalence (Methods; lines 204-213), this phrase is no longer used.

Please define “uncertainty interval”, do you mean “confidence interval”?

p.11 you write “certainty intervals”, do you mean “uncertainty intervals” or “confidence intervals”?

An explanation of how uncertainty intervals are defined has been added:

[Methods; lines 240-243]: “Each parameter is attributed with some uncertainty, either defined by a previously published uncertainty interval or based on a standard binomial distribution where the parameter is calculated from prior data. This uncertainty is propagated through subsequent calculations to obtain similar uncertainty intervals for sensitivity and FFI probability estimates.”

Avoid using “if” to introduce notation. Instead of writing “If<https://www.codecogs.com/eqnedit.php?latex=T%5E-#0> denotes …”, write “Let<https://www.codecogs.com/eqnedit.php?latex=T%5E-#0> denote …”.

The language has been changed as suggested:

[Methods; lines 163-4]: “Letting<https://www.codecogs.com/eqnedit.php?latex=T%5E-#0> denote the *observed* absence of infection and<https://www.codecogs.com/eqnedit.php?latex=I%5E-#0> the underlying *true* absence of infection, …”

The negative predictive value (NPV) is the conditional probability that there is a true absence of infection<https://www.codecogs.com/eqnedit.php?latex=I%5E-#0> given that the observed absence of infection<https://www.codecogs.com/eqnedit.php?latex=T%5E-#0>, i.e<https://www.codecogs.com/eqnedit.php?latex=NPV%20%3D%20P%5BI%5E-%7CT%5E-%5D#0>. The authors made a mistake and wrote:<https://www.codecogs.com/eqnedit.php?latex=NPV%20%3D%20P%5BT%5E-%7CI%5E-%5D#0>.

“NPV” is not an “analogy”, it is defined by<https://www.codecogs.com/eqnedit.php?latex=NPV%20%3D%20P%5BI%5E-%7CT%5E-%5D#0>

Thank you for flagging the error in the notation. This has been corrected and the reference to “analogy” removed:

[Methods; lines 163-166]: “Letting<https://www.codecogs.com/eqnedit.php?latex=T%5E-#0> denote the *observed* absence of infection and<https://www.codecogs.com/eqnedit.php?latex=I%5E-#0> the underlying *true* absence of infection, then the value of interest is the conditional probability<https://www.codecogs.com/eqnedit.php?latex=P%5BI%5E-%7CT%5E-%5D#0>. This is known as the negative predictive value (NPV), in the medical literature often interpreted for an individual diagnostic test, …”

State somewhere in the manuscript that Nigeria has 774 LGAs.

We have also now stated the number of LGAs in Nigeria at first reference:

[Methods; lines 147-9]: “We aggregated these data to a monthly time scale and a spatial scale of the local government authority (LGA; administrative level two) of which Nigeria has 774.”

For better clarity, the authors should define sensitivity and specificity also with conditional probability when they are introduced for the first time in the subsection “Statistical framework”

These definitions have been added:

[Methods: lines 173-180]:

<https://www.codecogs.com/eqnedit.php?latex=NPV%20%3D%20%5Cfrac%7BSp%20*%20(1-%20p)%7D%7BSp*(1-p)%20%2B%20(1-Se)*p%7D#0>

where

<https://www.codecogs.com/eqnedit.php?latex=Se%20%3D%20P%5BT%5E%2B%7CI%5E%2B%5D%20%3D%20%5Cfrac%7BTrue%5C%3Apositives%7D%7BTrue%5C%3Apositives%20%2B%20False%5C%3Anegatives%7D#0>

and

<https://www.codecogs.com/eqnedit.php?latex=Sp%20%3D%20P%5BT%5E-%7CI%5E-%5D%20%3D%20%5Cfrac%7BTrue%5C%3Anegatives%7D%7BTrue%5C%3Anegatives%20%2B%20False%5C%3Apositives%7D#0>

following the standard definitions of sensitivity and specificity.”

For greater precision, it should be specified that the link between NPV and Se, Sp is made by Bayes' formula

The role of Bayes’ theorem has been emphasised:

[Methods; 176-8]: Using Bayes’ theorem to invert the conditional probabilities, the NPV is linked to the sensitivity<https://www.codecogs.com/eqnedit.php?latex=Se#0> and specificity<https://www.codecogs.com/eqnedit.php?latex=Sp#0> of the test and to the underlying prevalence (<https://www.codecogs.com/eqnedit.php?latex=p#0>) of the condition being tested for:

When authors estimate the probability that an infected individual falls within the catchment area, are they assuming that the risk of infection is spatially homogeneous across the entire national population? If so, this assumption should be stated explicitly.

The assumption is now explicitly stated:

[Methods; lines 270-1]: “Assuming all under-15s in the LGA as independent and that risk of infection is homogenous within each LGA,...”

1/500 > 1/5000, so authors should write : « with 95% quantile interval between 1/5,000 and 1/500 ». A comma is missing for 1/5000, should be 1/5,000.

These grammatical errors have been corrected.

**Results :**

Define « IQR » before using it for the first time.

Definition of the acronym has been added:

[Results; lines 338-9]: “Although the median population size within 5km of a collected sample is 169,000 (Inter-Quartile Range; IQR [42,500 - 402,700])...”

In the abstract you write « In contrast, we conclude a probability of 98% (97.9-98.7%) by the time elimination of the serotype was officially declared in 2020. » whereas in the results you write : « the FFI probability that is attained by the official declaration of WPV elimination in August 2020 (41,42) is 98.2% (97.6-98.6%). » Why estimation of FFI at time of declaration of elimination does not fully correspond in the Abstract and Results ?

Apologies for this oversight - the values in the results had not been updated from a previous iteration to match the final results presented in the abstract. This has been corrected.

[Results; lines 428-430]: “...we find that the FFI probability that is attained by the official declaration of WPV elimination in August 2020 [(42,43)](https://www.zotero.org/google-docs/?CflHM9) is 98.2% (97.9-98.7%).”

**Figures/Tables:**

**Table 1:**

The heading “95 % certainty interval” should read “95 % uncertainty interval” (or “95 % confidence interval”).

This typing error has been corrected.

The confidence interval for the probability of notification is listed as 0.60–0.99, whereas the Supplementary Material gives 0.60–0.999. Please standardise. Note that 0.999 rounds to 1.00 (two decimal places), not 0.99.

Corrected in the main text to round to 1.00.

Subscripts “i” are missing for P^Clinical, P^StoolTest, P^SampleTest

The omission of subscripts is intentional as these values are not indexed by time or LGA.

**Figure 2:**

For greater clarity, please split the current Figure A into two panels and label them A and B (which means the present panel B will become C). In addition, state in the caption that ES = environmental samples.

This figure/caption has been amended as suggested:

***Figure 2:*** *Detections of wild poliovirus serotype 1 (WPV1) among cases of* ***(A)*** *AFP and* ***(B)*** *environmental surveillance (ES) samples. The total number of environmental samples collected is indicated by unfilled blue squares.* ***(C)*** *The recorded locations of four WPV1-positive cases of AFP detected in Borno state in July 2016, after 23 months of surveillance without detection (AFP cases are plotted randomly within LGA boundaries).*

**Reviewer 3**

This clearly written manuscript addresses a vexing epidemiological problem in a systematic and structured way. It is informed by previous modelling efforts and a real world context. The foundational epidemiological basis for the model is correct and the programmatic surveillance context and data are appropriately integrated into the model. The assumptions are valid and justified in the context of previous work. The findings are therefore robust and will contribute to the ongoing dialogue and recommendation development processes that are happening at a global level regarding cessation of bOPV2. This work should be published.

There are two possible areas for clarification, and a number of minor points.

**Re the numbers of people under environmental surveillance**

In the paragraph on page 14 (below Figure 3), the estimated population size within a 5km radius is 169,000. If the overall population density of Nigeria is 261/km2 (Wikipedia), and the area of a circle with radius 5km is 78km2, then the population within a 5km radius of the ES = 261x78=20,358 persons. If each ES site is designed to collect samples from c. 100,000 persons, this implies a population density of 1,282 persons/km2 within a 5km radius of each ES sampling site. This seems unrealistic.

We have further emphasised in the Methods (sub-section: Polio surveillance and data collection) that 100,000 is a target for design but doesn’t necessarily reflect reality:

[Methods; lines 137-140]: “Sites were initially selected with the intention to capture a population of approximately 100,000. In practice there is no guarantee that a site reflects this intended catchment size, which is likely to vary substantially depending on the positioning of the site and population movement since its initiation. We therefore estimate a catchment size for each site based on recent estimated population count within a fixed radius, instead of assuming that sites meet this target. This approach was shown by Hamisu et al. (5) to correlate more strongly with enterovirus prevalence (an indicator of catchment size) than estimates reported by ES officers.”

The figures of 169,000 persons within a catchment radius of 5km (on p 14, as above) appears to contradict the figures given in the discussion on page 20, paragraph 1 ‘For environmental surveillance….’ where by implication a LGA with a single ES site has only 12,500 people under surveillance.

We appreciate that the interpretation of these numbers is confusing. The point to note is that, across *all* sites that were sampled *at least once* during this time period, the average catchment within 5km was 169,000. This number also includes any population of neighbouring LGAs still within 5km. However, across all LGAs and months, the average proportion of population within 5km of a sampled site *and within that LGA* is much lower; this value excludes catchment outside the LGA of interest and - more importantly - includes months and LGAs in which no samples were collected (i.e. catchment of zero). The distinction between the numbers is therefore that the first is an average across all sampled sites, and the second is an average per month/LGA.

A further explanation of this distinction has been added:

[Discussion; lines 583-7]: This target is met by most collection sites when assuming a catchment radius of 5km. However, if we consider LGAs with either inconsistent sampling (i.e. some zero months) or no ES at all, only around 3% of an LGA’s population is captured on average per month across the country. This equates to approximately 12,500 people, increasing to 19,000 people between 2016-20.

Is it possible to use the actual numbers/proportions of persons under surveillance in Nigeria, rather than estimate them. I think actual numbers based on topographical and GIS data are available and used by the polio ES programme through Novel-T software (ES.world). The authors have stated in the discussion that ‘this work is yet to be adequately linked to sample locations’ – is this still the case amongst Nigerian ES sites?

The reviewer is correct that estimates of watershed areas based on DEM data do exist for certain catalogued ES sites. Hamisu et al (2022) demonstrate that the estimates appear to be reasonable for sites in Nigeria, but also find that they correlate with a simpler estimation of catchment based on a fixed radius. Our intention was for the framework to be applicable beyond this specific example, hence not to rely on estimates available for a limited number of locations. At the time of writing, the catalogue of sites did not cover the recorded locations of a majority of samples from Afghanistan and Pakistan, for example. We understand that a new version of the Novel-T platform has been published that allows watershed areas to be calculated for an arbitrary set of co-ordinates, however it would not be feasible to validate these new estimates and integrate the process into our workflow for this paper. In ongoing work we are exploring opportunities for validation in different settings and will consider incorporating them into future analyses of ES data.

**Re the application of the model in other contexts**

Would it be illustrative to take the model a step further by serially halving and serially doubling (or decreasing/increasing by some other reasonable factor) the surveillance performance data inputs and determining how this impacts on the probability of correctly determining FFI in the Nigerian context?

We would argue that this would only be informative if it were in reality feasible to halve/double performance of the surveillance system, which is unlikely. The potentially modifiable components of the system would be coverage of ES and notification of AFP (stool sample quality is already quite high and there would be no intentional reason to reduce it, e.g. for economising). A more relevant comparison would be between *observed* differences in these components across countries. Such an analysis was conducted to look at cVDPV2 elimination between several countries with periods of observed absence (Cote D’Ivoire, South Sudan, DRC) and was presented to the GCC in November 2024 (See [this report](https://polioeradication.org/wp-content/uploads/2025/04/26th-meeting-of-the-Global-Commission-for-the-Certification-of-Eradication-of-Poliomyelitis-Final.pdf)). This demonstrated how more sparse environmental sampling in South Sudan results in much slower accumulation of confidence in elimination.

How exactly may the model be applied in real-world contexts at national level particularly with regard to declaring a VDPV2 outbreak over. How would the required data differ, or how could the model be strengthened to make it more context-specific?

A section on the application of this framework to cVDPV2 outbreaks has been added to the discussion:

[Discussion, Implications for future research; lines 608-617]:We suggest that this approach may also be applied to interpret the resolution of vaccine-derived poliovirus (VDPV) outbreaks, in particular those of type 2 (cVDPV2) which became widespread following the removal of serotype 2 from standard oral vaccination [(55)](https://www.zotero.org/google-docs/?G2kpBJ). Lower symptomatic rates for serotype 2 would mean that AFP surveillance would be substantially less sensitive for cVDPV2 relative to ENV surveillance. This means that greater effort invested in consistent and well-distributed environmental sampling would be necessary to declare with confidence that a cVDPV2 outbreak is over. Such an analysis was conducted and presented to the GCC in November 2024 [(56)](https://www.zotero.org/google-docs/?7Si1xK), highlighting the sparse coverage of environmental surveillance in several countries currently experiencing cVDPV2 outbreaks, relative to the coverage in Nigeria for the near-WPV1-elimination period presented here.

What does the model tell us about the WP1 outbreak in Mozambique/Malawi in 2021-2? Is it over? Or could we expect WP1 to be circulating in the region today?

The objective of this paper is to explain and validate the methodology for a historical example in which we know the outcome. It would definitely be valuable to apply the approach to the suggested example, but is beyond the scope of this specific paper.

**Other minor comments/thoughts**

A semantic/nomenclature type question – the ‘design prevalence’ (in the section ‘Freedom from Infection’) implies that the polio surveillance system, comprising AFP and ES data has been ‘designed’ to meet a specific prevalence requirement that is required to interrupt transmission. Would it not be more correct to call it the ‘System prevalence’ or the ‘Minimum detectable prevalence’? If I understand it correctly – it is the lowest prevalence which the system is capable of detecting? The name ‘design prevalence’ seems tautologous, given that the sensitivity of the surveillance system could not be designed when it was envisaged and set up.

We understand the reviewer’s point here about the term being not completely intuitive, however we chose this as it is consistent with the original presentation of this methodology (see *Martin et al. 2006*) and previous applications (e.g. *Watkins 2009*, *Stresman 2017*).

LGA is used for the first time without defining the abbreviation in the section ‘Freedom from infection’ (it is defined later in the text)

Thank you for flagging this omission. The acronym has now been defined at first use:

[Introduction, line 104]: “...in any local government authority (LGA, equivalent to district)”

Is there a basis for setting the ‘design prevalence’ at the level of 1/774 for the country, and for starting with the estimate of 1/100,000 ? is there evidence to suggest this? Given the discussion point that the conclusions are highly sensitive to the ‘design prevalence’, it may by helpful to explain the values the models started with – if it is possible to do so, without creating a circular argument (ie using the model outcome to justify the starting point!)

Please refer to our response to the first major comment from Reviewer 2 above.

The estimates in table 1 are not referenced.

The table is referenced in the following lines:

[Methods; lines 240-2]: “A detailed explanation of how the probabilities for each event were specified is given in the *Supplementary Materials*, and a summary is provided in *Table 1*.”

In the para ‘FFI probability over time’, the second paragraph begins with the word ‘Specifically’. I think this word is used for its linguistic meaning here, rather than its statistical meaning. So it may read better (in terms of meaning) to start with ‘Therefore…’ or some other way of transitioning to a more detailed explanation.

The word has been replaced as suggested:

[Methods; line 311]: “Therefore, the probability of freedom from infection in month *t+1…”*

Map D in Figure 4 could be enlarged for easier readability

Maps in figures 3 and 4 have all been enlarged.

In the Discussion, in the section on ‘our estimation of AFP sensitivity’ – patient level (care-seeking) and health system level factors will impact the probability of a symptomatic case being reported. Conflict and social instability may impact both factors. In these settings, could all-cause mortality rates in U15s provide some context for interpreting or moderating the probability of AFP detection?

This is an interesting suggestion. After consideration we would argue that, although lower all-cause mortality in U15s would likely correlate with better care-seeking and access, it would be challenging to translate this to a *probability* of AFP detection. Unlike the AFP notification rate, for which we have an expert-informed WHO threshold to measure against, it is unclear what rate of all-cause mortality could be interpreted as indicating “adequate” care-seeking for detecting cases of AFP. As explained in the discussion, it should be a focus of future work to investigate how reported levels of conflict and instability between regions are associated with different indicators of care-seeking/access, such as AFP notification and childhood all-cause mortality rates. With this understanding, it may be possible to incorporate other indicators of care-seeking into this model framework in the future.

In the Discussion - the para starting ‘Having ES within sewage treatment works…’ can be contextualised by the fact that LMICs, and areas with highest risk for polio emergence have minimal wastewater infrastructure.

The comment about bias from sewage connections has been expanded to also reference the LMIC context:

This bias would be expected in most polio-affected settings in which this framework could be applied [(52)](https://www.zotero.org/google-docs/?rbOz5F) and, moreover, many of these are low-income settings with limited sewage infrastructure to begin with. Especially in informal sewage systems, the water flow, dilution, temperature, chemical contamination and recent weather will also influence what is detected in a collected sample [(53,54)](https://www.zotero.org/google-docs/?oEAOul).
