# Supplementary material for: Sub-national modelling of surveillance sensitivity to inform declaration of disease elimination: A retrospective validation against the elimination of wild poliovirus in Nigeria
Source: PLoS Comput Biol. 2026 Mar 16;22(3):e1013984. doi: 10.1371/journal.pcbi.1013984 (PMC13035237; doi:10.1371/journal.pcbi.1013984)
Supplement: Attachment — Submitted filename: Review2.docx [file pcbi.1013984.s005.docx]

Review (second round) the manuscript: « Sub-national estimation of surveillance sensitivity to inform declaration of disease elimination: A retrospective validation against the elimination of wild poliovirus in Nigeria »

Submitted in POS Computational Biology

Manuscript number: PCOMPBIOL-D-25-00277

**Summary**

Authors answered carefully to the comments. I recommend publication.

**Typos**

**Material and methods:**

- The sentence “We specify the design prevalence at two levels: LGA and country” is repeated twice.

**Figures/Tables:**

- Table 1: In column Event, there are twice the math symbol of the parameter.
- Figure S7: in the legend there is a missing space “α=30and”.
