# Supplementary material for: Sub-national modelling of surveillance sensitivity to inform declaration of disease elimination: A retrospective validation against the elimination of wild poliovirus in Nigeria
Source: PLoS Comput Biol. 2026 Mar 16;22(3):e1013984. doi: 10.1371/journal.pcbi.1013984 (PMC13035237; doi:10.1371/journal.pcbi.1013984)
Supplement: Attachment — Submitted filename: Response to reviewers - 0601.docx [file pcbi.1013984.s007.docx]

Faculty of Epidemiology and Population Health

London School of Hygiene and Tropical Medicine

Keppel Street, London, WC1E 7HT, U.K.

E-mail: emily.nightingale@lshtm.ac.uk

6^th^ January 2026

*Plos Computational Biology*

Dear Editor,

**RE: “Sub-national estimation of surveillance sensitivity to inform declaration of disease elimination”**

We’d like to thank the reviewer for their positive feedback on our revised manuscript and for highlighting these minor typing errors, which have been resolved in the attached version.

Following our exchange in September regarding the use of copyrighted shapefiles, note that this version has figures amended to use shapefiles from a public source. This source is now acknowledged in the caption of each relevant figure.

Since submitting the last revision, we identified a minor error in the definition of one of the surveillance indicators used in our model. We incorrectly stated that the sensitivity indicator used by the WHO for environmental surveillance was the detection rate of *non-polio* enteroviruses in collected samples, whereas it should be the detection rate of *any* enteroviruses. This is a minor component of the model and amending the definition made negligible difference to our calculated estimates and no difference to our conclusions. The attached revision reflects the corrected definition.

Kind Regards,

Emily Nightingale
